# Inflammasome is a central player in B cell development and homing

**DOI:** 10.26508/lsa.202201700

**Published:** 2022-11-30

**Authors:** Man Lun Hsu, Moncef Zouali

**Affiliations:** Graduate Institute of Biomedical Sciences, China Medical University, Taichung, Taiwan

## Abstract

Whereas the role of the nucleotide-binding oligomerization domain, leucine-rich repeat and pyrin domain–containing protein (NLRP) 3 pathway in innate immunity has been extensively studied, little attention has been paid to its contribution to adaptive immunity. Studies in animal models and human subjects have shown the contribution of NLRP3 to the T cell compartment, and its role in B lymphocyte functions has been proposed. Here, we report that ablation of *nlrp3* in mice led to altered B cell development in the bone marrow, and distorted expression of B cell subsets that play innate-like functions, that is, marginal zone B cells in the spleen and B-1a cells in the peritoneal cavity. Mechanistically, in the absence of NLRP3 expression, the transcription factor IRF4, previously found to interact with NLRP3 in the nucleus of lymphocytes, was up-regulated. NLRP3 ablation reduced the expression of the chemokine receptors CXCR4 and CCR7 in an IRF4-dependent manner, indicating that the presence of NLRP3 is critical for optimal expression of chemokine receptors on B cells. We conclude that activation of the NLRP3 inflammasome plays a role in B cell development, homing, and retention in lymphoid organs.

## Abstract (precis)

Whereas the inflammasome is known to be a key player in innate immunity, this study shows that NLRP3 is essential for egress/mobilization of B cells from the bone marrow to secondary lymphoid organs.

## Introduction

B lymphocytes develop from hemopoietic stem cells in the bone marrow (BM) to mature B cells in the periphery through sequential checkpoints (Melchers, 2015). Their development is a highly ordered process that begins with pro-B cells that initially progress to the pre-B cell stage. Upon expression of a functional pre-B cell receptor (BCR), pre-B cells proliferate and undergo rearrangement of their Ig κ and, then λ, light chain (L) genes to differentiate into immature B cells carrying a BCR of the IgM isotype on their surface. After expression of cell surface Ig, IgM+ immature B cells leave the BM and migrate to peripheral lymphoid organs where they progress to the mature B cell stage and acquire marked phenotypic changes, giving rise to transitional type 1, and, then, transitional type 2 B cells. This latter cell subset progresses to mature follicular (FO) B cells defined as IgM^low^IgD^high^CD23^high^CD21^+^ (Martin et al, 1995). In addition to this recirculating B cell subset, the spleen includes a population of mature marginal zone (MZ) B lymphocytes (IgM^high^ IgD^low^CD23^low^CD21^high^) that reside in the MZ and represent ∼10% of the B cell splenic population of the mouse. In contrast to FO B cells, MZ B cells do not recirculate and exhibit distinct functional properties, such as rapid activation and differentiation into Ab-secreting cells upon stimulation with various ligands. In addition, MZ B cells usually express germline-encoded BCRs exhibiting autoreactive and/or antimicrobial specificities (Martin et al, 2001).

Reminiscent of the functions of MZ B cells are B-1a cells that express the CD5 marker and exhibit a self-renewing capacity. This cell subset is barely detectable in the spleen but enriched in the coelomic (peritoneal and pleural) cavities of the mouse (Zouali & MacLennan, 1992; Hardy & Hayakawa, 2001). In addition to being anatomically isolated from the other cell types, MZ and B-1a cells share various functional similarities, such as high antigen-presenting capacity (Martin et al, 2001) and preferential secretion of complement-fixing, multi-reactive Igs called natural Abs (Ochsenbein et al, 1999). The secreted Igs are generally germline encoded and exhibit a reduced length of non-template N-additions in variable region genes. This Ab subset plays an important role in protection against microbial infections (Baumgarth et al, 2000). Upon in vitro stimulation with LPS, anti-IgM Abs, or CD40 ligands, MZ B cells have been shown to proliferate rapidly and vigorously for more prolonged time periods than FO B cells and secrete copious amounts of Igs. Thus, because of their anatomical location and their functional properties, B-1 and MZ B cells are involved in T cell–independent innate–like immunity and represent an immune mechanism of first-line defense (Viau & Zouali, 2005).

In response to microbial infection or cellular damage, a group of protein complexes, that is, the inflammasomes, form in the cytoplasm to mediate host immunity. Their assembly leads to proteolytic cleavage of procaspase-1 into active caspase-1, which triggers conversion of the cytokine precursors pro-IL-1β and pro-IL-18 into biologically active cytokines that act as potent pro-inflammatory mediators in several immune reactions, such as the recruitment of cells important for innate immunity and modulation of the adaptive branch of immunity (Martinon et al, 2009; He et al, 2016; Swanson et al, 2019). Given the potential involvement of NLRP3 in several human diseases, this inflammasome has been the focus of much investigation. NLRP3 has been primarily studied in cells of the innate immune system, that is, monocytes, macrophages, granulocytes, and dendritic cells, but, more recently, in T and B lymphocytes (Bruchard et al, 2015; Arbore et al, 2016; Ali et al, 2017). In view of the multiple roles of B cells in innate immunity, we undertook the present investigation to determine the effects of deleting the *nlrp3* gene from the mouse genome on B cell development. The approach taken led to unexpected novel findings. Significantly, we found that lack of NLRP3 resulted in distorted expression of B cells exhibiting innate-like functions.

## Results

To investigate the function of NLRP3 in B lymphocytes, we assessed B cell development in C57BL/6 mice lacking nlrp3, as compared with WT mice.

### Light chain allelic exclusion in NLRP3^−/−^ mice

**Figure S1.**
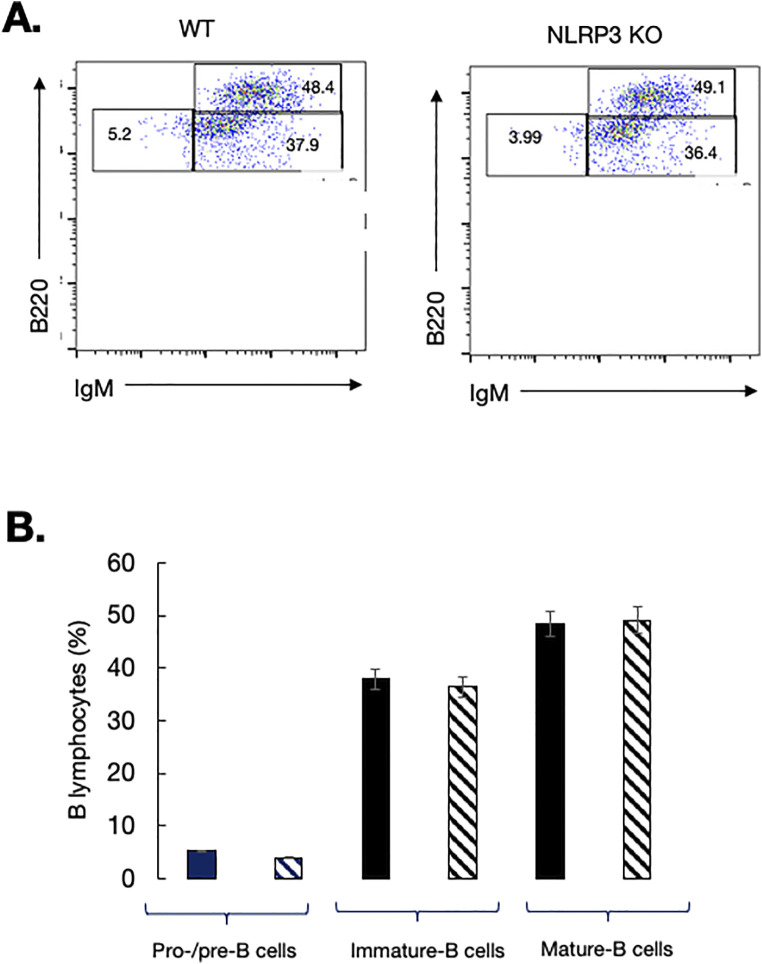
B cell development in the bone marrow of NLRP3-deficient mice. **(A)** B cells at different developmental stages can be defined by the expression of B220 and IgM surface markers. Single-cell suspensions were labeled with the indicated Ab combinations for flow cytometry analysis. Percents of positive cells within quadrants are indicated. B220^low^IgM^−^ cells are B cell precursors including pro/pre-B; B220^low^IgM^+^ are immature B cells, and B220^high^IgM^+^ are recirculating mature B cells. **(B)** WT mice: black bars; NLRP3^−/−^ mice: hatched bars. Data are representative of five individual mice of each genotype.

In the BM, we analyzed B cell subsets that included pro-B and pre-B (B220^+^IgM^−^) and immature (B220^+^IgM^+^) and mature (B220^hi^IgM^+^) B cells as described (Viau et al, 2005). Generation of pro/pre-B cells and immature B cells was normal in the BM of mutant mice. The size of the mature B compartment was also comparable to that of control mice (Fig S1). Thus, lack of NLRP3 does not alter significantly early B cell development in the BM.

**Figure 1.**
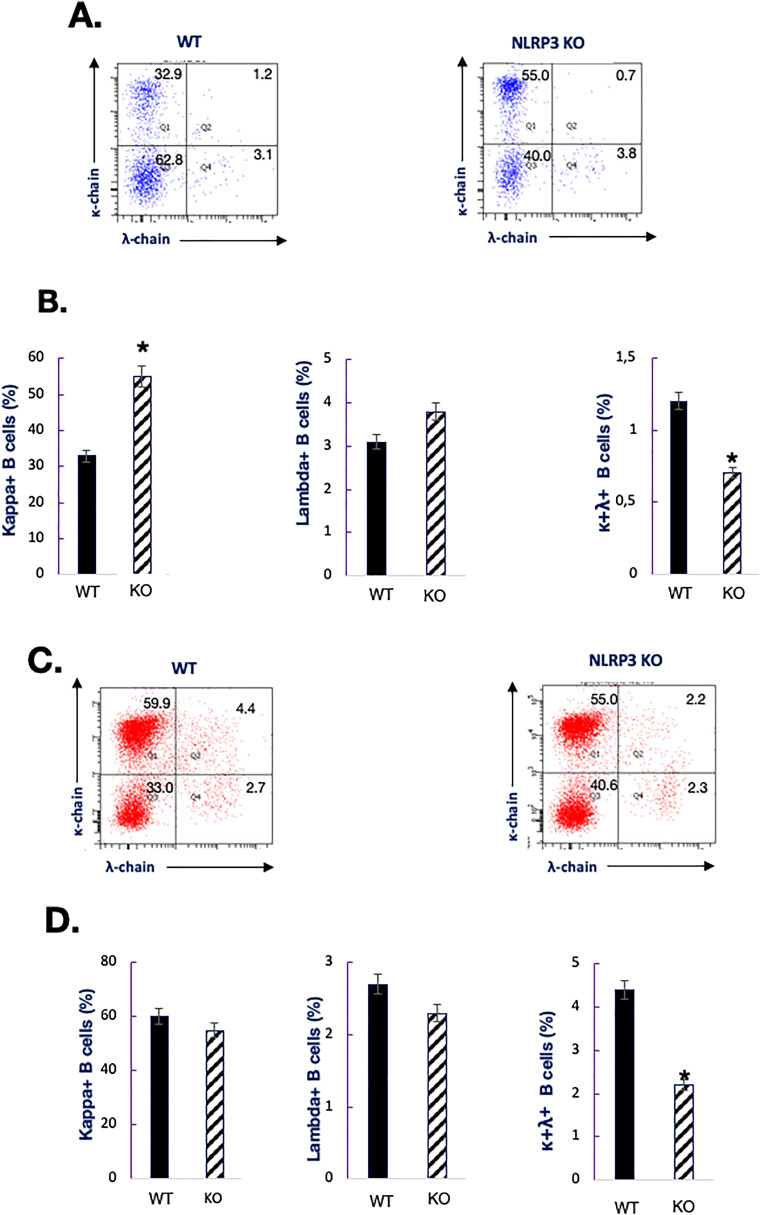
Analysis of allelic exclusion in NLRP3^−/−^ mice. Single-cell suspensions were simultaneously labeled with anti-Igκ antibodies and anti-Igλ antibodies. Stained cells were analyzed by flow cytometry. Percentages of positive cells residing in different windows are shown in the quadrants. Data are representative of five individual mice of each genotype. (*): *P* < 0.05. **(A, B)** FACS analysis of cell surface Ig expression in the bone marrow. **(C, D)** FACS analysis of cell surface Ig expression in the spleen.

Throughout their development, B cells express surface heavy (H)- and L-chains encoded by a single allelic copy of the corresponding locus, a process called antigen receptor allelic exclusion (Vettermann & Schlissel, 2010). We asked whether NLRP3 may specify allelic exclusion in the B cell compartment. To test the possibility that NLRP3^−/−^ mice exhibit allelic inclusion for L-chain loci, we assessed kappa-positive (*κ*) and lambda-positive (*λ*) B cells. FACS analysis showed that NLRP3-deficient mice exhibited higher levels of usage of the mouse Cκ exon in BM tissues compared with NLRP3-sufficient mice (Fig 1A and B). We then evaluated L-chain inclusion of Ig*κ* and Ig*λ* loci in control and NLRP3^−/−^ mice by FACS analysis. As shown in Fig 1C and D, ablation of *nlrp3* resulted in decreased percentages of double-positive *κ*^+^ and *λ*^+^ B cells, in both splenic and BM tissues. We conclude that NLRP3 interferes with allele usage for the Ig*λ* locus and L-chain allelic exclusion in the B cell compartment.

### Expression of transcriptional activators in B cells of NLRP3 mutant mice

**Figure 2.**
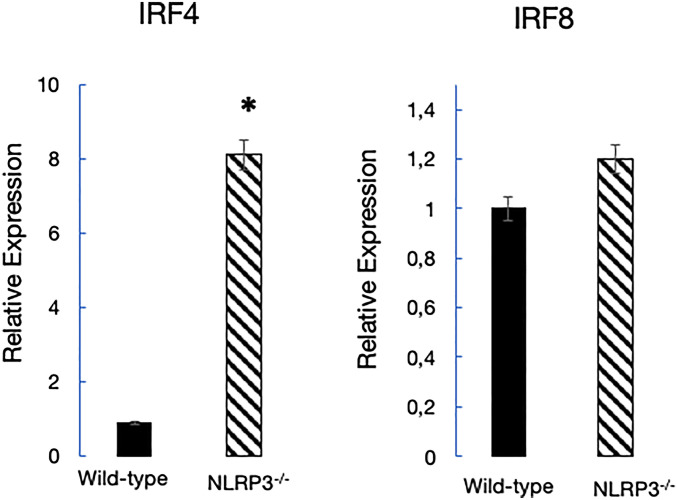
NLRP3 deficiency leads to overexpression of IFR4 in B lymphocytes. B cells isolated from WT and mutant mice were cultured for 3 d, and the relative mRNA expression level of IRF4 and IRF8 was determined as described in the Materials and Methods section. The results represent cumulative data obtained in different single experiments. (*): *P* < 0.05.

Because λ-expressing B cells are frequently considered as products of receptor editing and secondary rearrangements (Radic & Zouali, 1996), the distorted *κ*/*λ* ratio in the BM of mice lacking NLRP3 could result from altered receptor editing in the mutant mice we have analyzed. Previous studies showed that pre-B cell development is under the control of the transcriptional activators IRF4 and IRF8, known to promote L-chain rearrangement and transcription (Xu et al, 2015). We therefore sought to determine if NLRP3 deficiency impacts expression of IRF4 and IRF8. As an approach to addressing this question, we quantified IRF4 and IRF8 levels in purified B cells. Relative levels of mRNA expression of IRF4 and IRF8 were determined by quantitative real-time polymerase chain reaction (qRT–PCR) and quantified by the 2^−ΔΔC^T method. We found that the relative levels of IRF4 expression were higher in mutant mice, as compared with WT rodents, and that the IRF8 levels were similar for both mouse strains (Fig 2). These results suggest that the distorted *κ*/*λ* ratios in NLRP3^−/−^ mice are due to up-regulated expression of IRF4.

### Alterations of mature B cell subsets in the periphery of NLRP3^−/−^ mice

**Figure 3.**
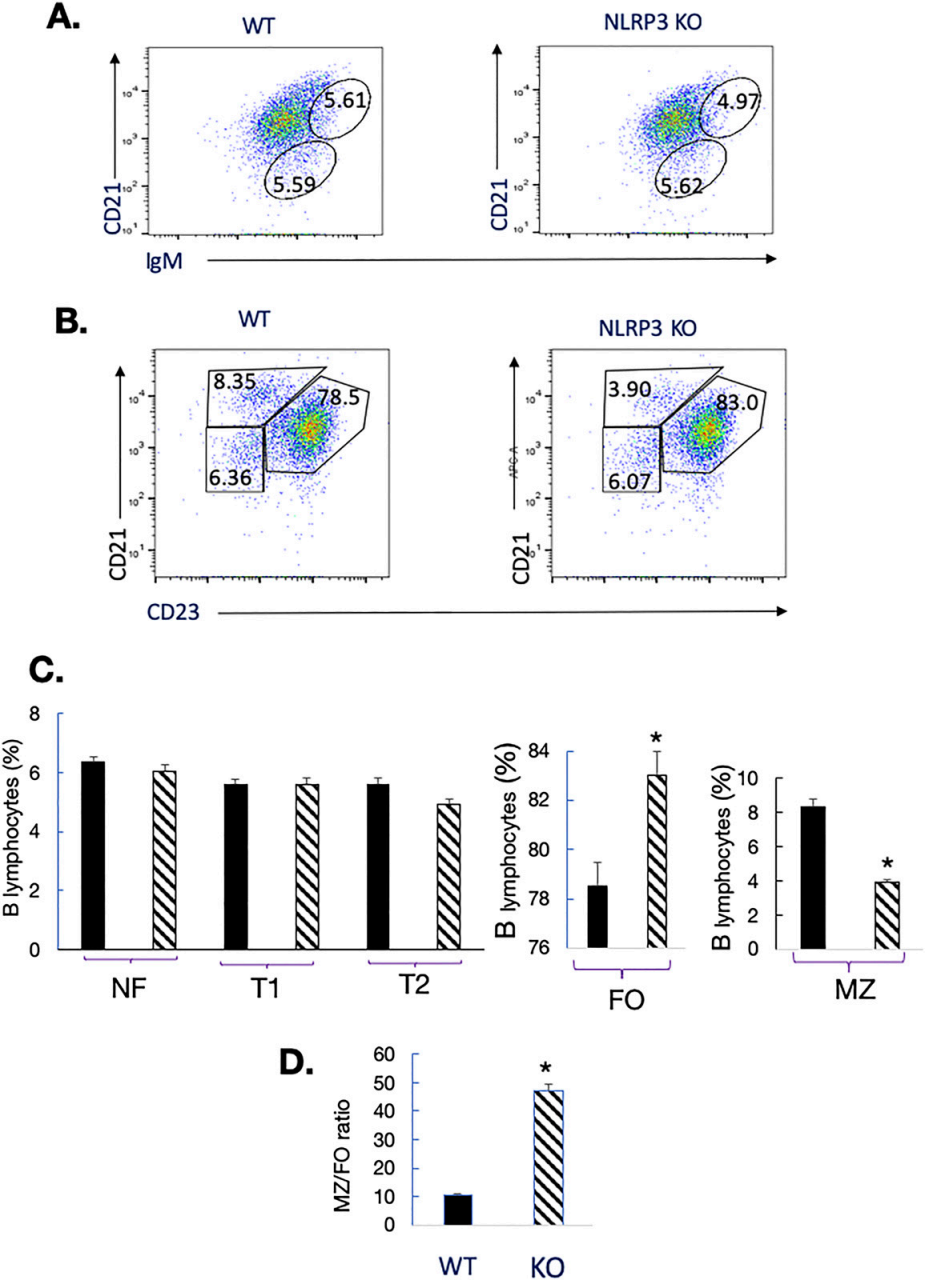
Reduction of marginal zone B cells (MZ) in the spleen of NLRP3^−/−^ mice. Single-cell suspensions of spleens from C57BL/6 and mutant mice were generated and analyzed by flow cytometry. A minimum of 10,000 events were collected per sample. Low angle and orthogonal light scatters were used to exclude dead cells and debris. Cells with the forward and side light scatter properties of lymphocytes were analyzed with fluorescence intensity shown on a four-decade log scale. Positive and negative populations of cells were determined using unreactive isotype-matched antibodies as controls for background staining. **(A)** Shown are representative dot plots of newly formed NF B cells (B220^+^CD23^−^CD21^−^); transitional T1 (B220+IgM^high^CD23^low^); and transitional T2 (B220+IgM^high^CD23^high^). Percentages of positive cells in C57BL/6 and KO mice are displayed in the corresponding quadrants. **(B)** Spleen cells from WT or KO mice were stained with fluorescently labeled anti-CD21, anti-CD23, and anti-B220 antibodies, and cells were gated on B220 expression. Shown are representative dot plots of follicular FO B cells (B220+CD21^low^CD23^high^) and MZ B (B220+CD21^high^CD23^low^). The percentages of positive cells in C57BL/6 and KO mice are displayed in the corresponding quadrants. **(C)** Percentages of NF, T1, T2, FO, and MZ B cells in the spleen of WT (black bars) and mutant mice (hatched bars) are indicated. Bars represent the mean value for five mice per group, and error bars represent the SD. **(D)** FO/MZ ratios in the spleen of WT (black bars) and mutant mice (hatched bars). (*): *P* < 0.05. The FO/MZ ratios represent the ratios of the percentage of FO B cells/the ratios of the percentage of MZ B cells in the spleen of the mice analyzed.

The distribution of B cell subsets in the spleen of NLRP3^−/−^ mice was analyzed by FACS (Fig 3A and B). In mutant mice, there was an expansion of FO B cells associated with an over 50% reduction of MZ B cells (from 8.2% in WT to 3.8% in NLRP3 KO mice). Because NLRP3-deficient spleens harbored slightly fewer B cells (61.8%) than WT (67.7%), the alterations seen in FO B cells (that constitute the most abundant B cell population in the spleen) do not reflect the decreased absolute B cell numbers. Thus, even though NF B cell numbers were normal in NLRP3 KO mice, marked changes targeted the two mature splenic B cell subsets, with decreased MZ B cells but increased FO B cells. These alterations, which resulted in changes in the splenic FO/MZ ratio (Fig 3C and D), cannot account for the 9% overall reduction of B cells in the spleen of NLRP3-deficient mice (61.8%), as compared with WT rodents (67.7%).

### Expansion of B-1a cells in the peritoneal compartment of NLRP3-deficient mice

**Figure 4.**
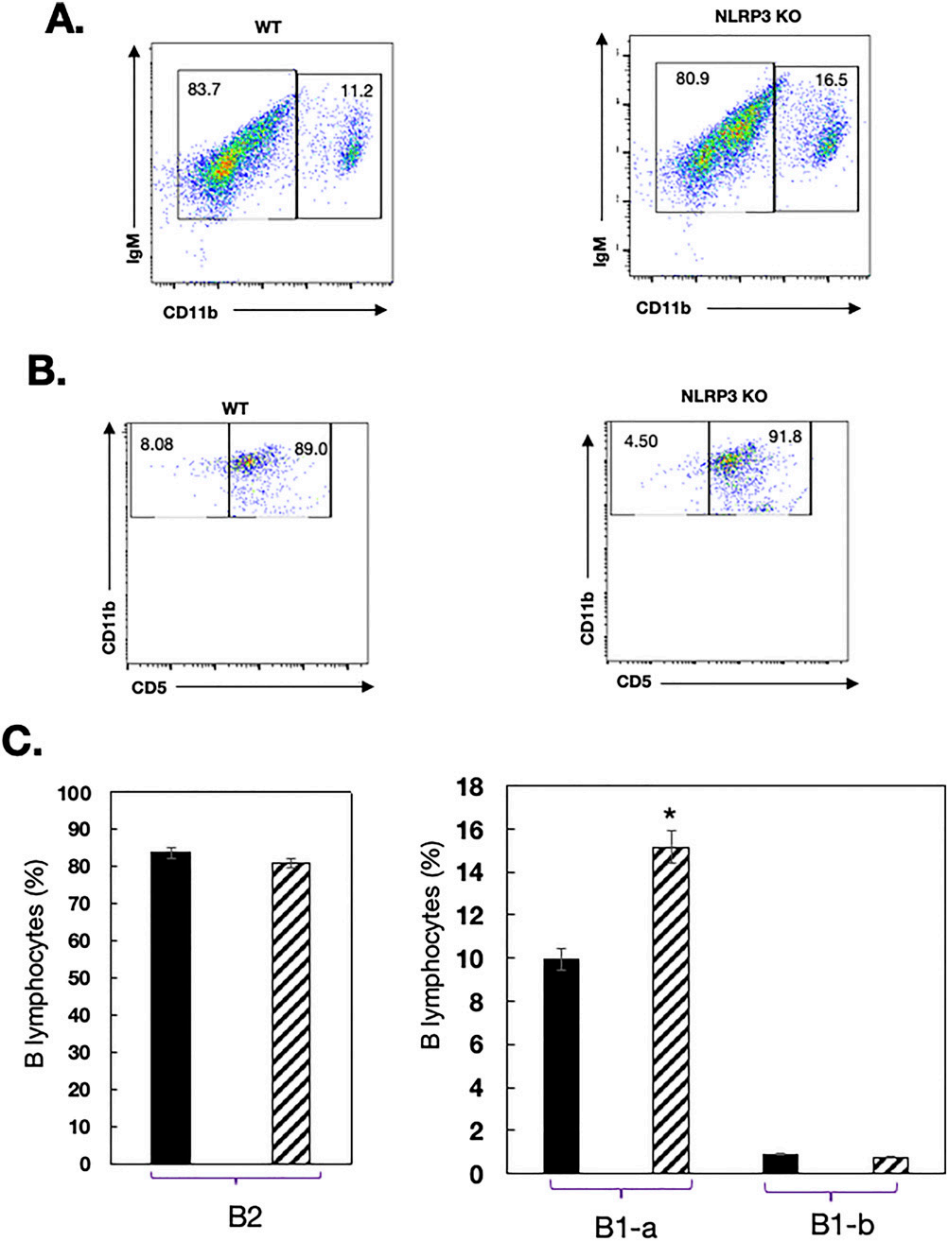
Expansion of B-1a cells in the peritoneal cavity of NLRP3^−/−^ mice. Cells were obtained from peritoneal lavage of C57BL/6 and KO mice. Single-cell suspensions were labeled with the indicated Ab combinations for flow fluorescence analysis, and representative FACS profiles of B cell subsets are shown. **(A)** The percentages of B-2 cells (IgM^+^CD11b^−^) and B-1 cells (IgM^+^CD11b^+^) are displayed in the corresponding quadrants. Data are representative of four individual mice of each genotype. **(B)** The percentages of B-1a cells (IgM^+^CD11b^+^CD5^high^) and B-1b cells (IgM^+^CD11b^+^CD5^−^) of C57BL/6 and KO mice are displayed in the corresponding quadrants. Data are representative of four individual mice of each genotype. **(C)** The percentages of B-2, B-1a, and B-1b cells in the peritoneal cavity of WT (black bars) and mutant mice (hatched bars) are indicated. Bars represent the mean value for four mice per group, and error bars represent the SD.

Body cavities comprise conventional B-2 cells (CD5^−^CD11b^−^B220^hi^IgM^lo^) that are replenished by mature recirculating B cells, and the pool of B-1 cells (CD5^+^CD11b^+^B220^lo^IgM^hi^) that develop essentially from fetal liver precursors and is maintained by constant self-renewal (Ghosn et al, 2019). Here, we observed a discernible change in the peritoneal B cell compartment of NLRP3 KO mice. Whereas the number of B-2 cells in the peritoneal cavity of mutant mice was virtually normal, the size of the B-1 cell population was expanded compared with that of WT rodents (Fig 4A). Notably, there was a shift of the Cd11b-negative population toward the region defined in the WT scatter for the positive one. It is possible that this fluorescence shift results from a higher expression of cell markers recognized by the florescent Ab used. As shown in Fig 4B and C, the B-1 expansion was marked for the B-1a subset (from 9.96% of B220+ cells in normal mice to 15.14% in deficient mice). As a result, the B-1a/B-1b ratio underwent a marked increase, from 11.06 in normal mice to 20.45 in mutant mice. These observations suggest that NLRP3 is involved in regulating the development of B-2 and B-1 cells in the peritoneal cavity.

### Natural Ab production in NLRP3-deficient mice

**Figure S2.**
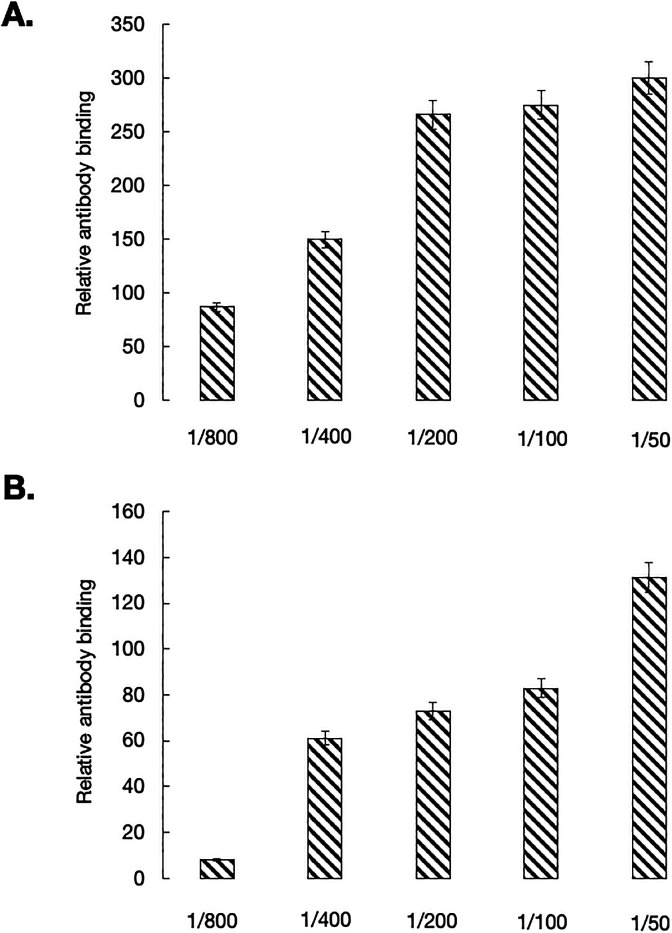
Reactivity of serum IgM from NLRP3-deficient mice with nuclear antigens. **(A, B)** Serum dilutions from C57BL/6 mice and mutant mice were tested for binding with eukaryotic antigens (A) and single-stranded DNA (B) by ELISA. Shown are the relative binding reactivities: (OD of serum from NLRP3^−/−^ mice) − (OD of serum from WT mice)/(OD of serum from WT mice) × 100.

In contrast to conventional B-2 cells, B-1a cells largely produce circulating natural Abs, that is, predominantly polyreactive, low-affinity IgM that also bind autoantigens and serve in the clearance of apoptosis products (Baumgarth et al, 2000). The expansion of B-1a cells we found in mutant mice prompted us to ask whether the NLRP3-deficient mice also exhibit increased serum levels of natural Abs. We therefore tested the binding reactivity of serum IgM from WT and mutant mice to single-stranded DNA and a set of soluble cytoplasmic and nuclear eukaryotic antigens. As shown in Fig S2, mice lacking NLRP3 had increased amounts of natural Abs, as compared with WT controls. These observations suggest that the absence of NLRP3 in C56BL/6 mice resulted in a B-1a cell expansion associated with an increased production of natural Abs.

### Proliferation capacity of B cells lacking NLRP3

**Figure S3.**
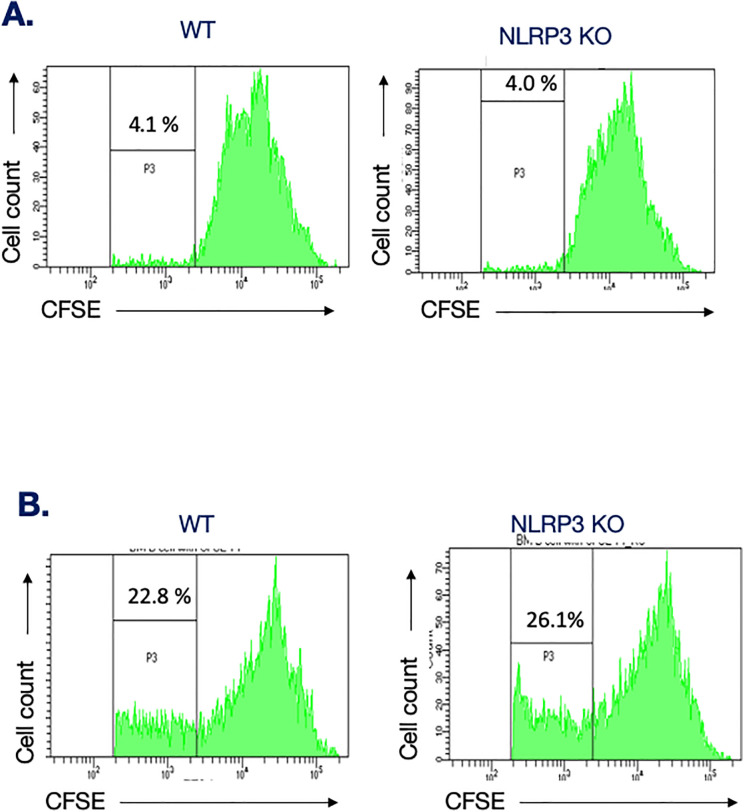
Proliferative response of B cells lacking NLRP3. CFSE-labeled B cells isolated form C57BL/6 mice and NLRP3-deficient mice were stimulated with PMA and ionomycin or left untreated. At 72 h, B cell proliferation was determined by FACS analysis of CFSE dye dilution ex vivo. **(A, B)** Shown are representative proliferation profiles of B cells incubated with vehicle (A) or a combination of PMA and ionomycin (B).

It is likely that humoral immunity to pathogens or self-antigens is associated with necrotic and apoptotic cells that can trigger a combination of T cell–dependent and –independent responses. Thus, we asked whether NLRP3 could play a role in humoral responses elicited in situations of strong cross-linking. This possibility was tested by stimulating ex vivo purified B cells in conditions mimicking strong B cell activation. As expected, a combination of the calcium ionophore ionomycin and the pleiotropic PKC activator PMA drove WT B cells to proliferate, as assessed by CFSE dye dilution (Fig S3). In parallel, the proliferative capacity of B cells isolated from NLRP3-deficient mice was not significantly affected, suggesting that NLRP3 does not play a significant role in the B cell proliferative capacity.

**Figure S4.**
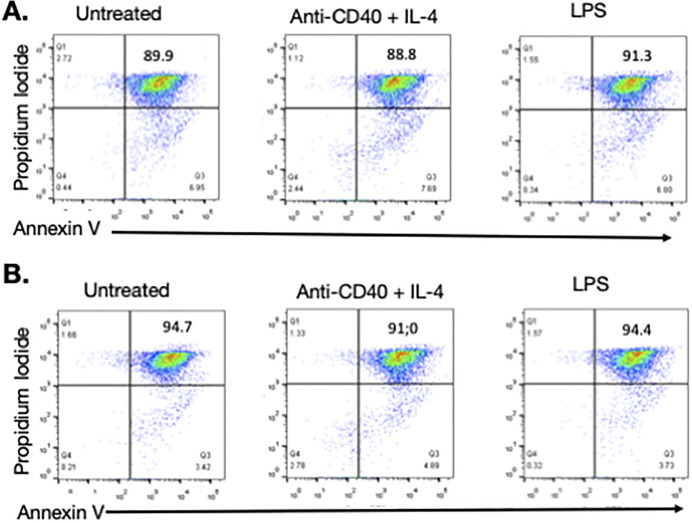
Apoptotic death of B cells in NLRP3^−/−^ mice. **(A, B)** B cells isolated from the spleen of WT (A) and mutant mice (B) were activated ex vivo in the presence LPS or a combination of anti-CD40 Ab and IL-4. After 3 d in culture, cells were stained with annexin V and propidium iodide, and cell death was examined by FACS analysis.

Given the increase in natural Ab levels, we asked whether lack of NLRP3 is accompanied with altered cell death that would lead to the release of antigens that could trigger B cell activation. Quantification of apoptotic cells by annexin V/propidium iodide staining revealed that apoptotic cell numbers were similar in NLRP3−/− and WT B cells analyzed after exposure to potent in vitro stimulation (Fig S4). In these experiments, control B cells exhibited a relatively high rate of apoptotic cell death. This could be because of the relatively long incubation periods of the cells. However, since apoptosis was not increased in mutant B cells, we conclude that lack of NLRP3 does not lead to an acceleration of B cell death and is not associated with increased proliferation.

### Chemokine receptor expression in mice lacking NLRP3

**Figure S5.**
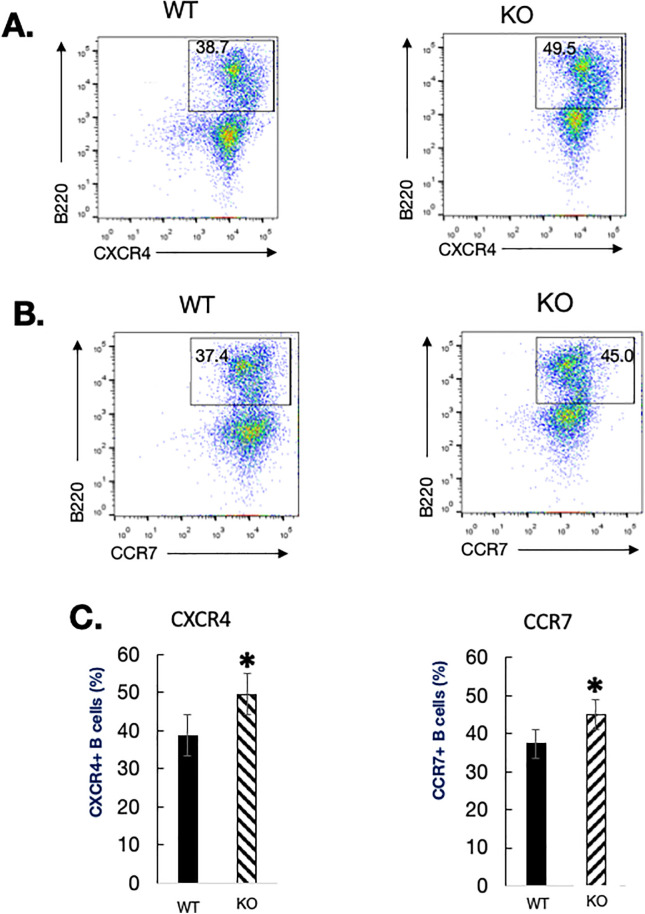
Chemokine receptor expression in B cells from NLRP3^−/−^ mice. **(A, B)** Single-cell suspensions were isolated from WT and mutant mice and simultaneously labeled with anti-CXCR4 and anti-B220 antibodies (A) or anti-CCR7 and anti-B220 antibodies (B). Stained cells were analyzed by flow cytometry. Percentages of positive cells residing in different windows are shown in the quadrants. **(C)** Percentages of CXCR4-positive and CCR7-positive B cells in WT (black bars) and mutant mice (hatched bars) are indicated (C). Bars represent the mean value for > 3 mice per group, and error bars represent the SD. (*) *P* < 0.05.

**Figure 5.**
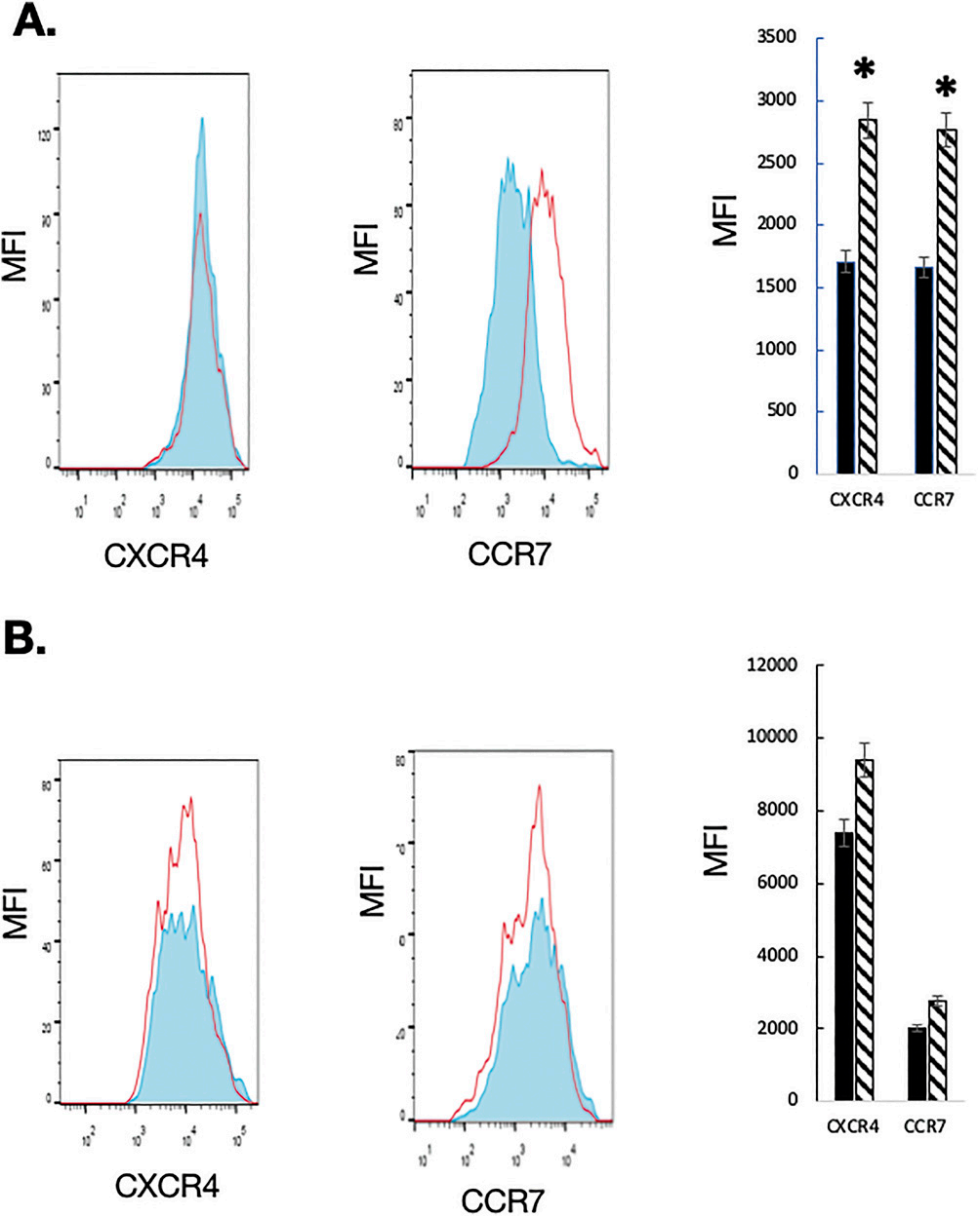
Chemokine receptor expression in B cells from NLRP3^−/−^ mice. **(A, B)** Single-cell suspensions were isolated from the BM (A) and the peritoneal cavity (B) of WT (shown in red) and mutant mice (shown in blue) and simultaneously labeled with anti-CXCR4 and anti-B220 antibodies or anti-CCR7 and anti-B220 antibodies. Stained cells were analyzed by flow cytometry (n = 4). The mean fluorescence intensity of CXCR4 and CCR7 expression by B lymphocytes is shown for WT (black bars) and NLRP3 KO mice (hatched bars). (*): *P* < 0.05.

The altered B cell homing and/or positioning of B cells in the absence of NLRP3 could be related to impaired chemotaxis of B cells to lymphoid organs. To clarify whether NLRP3 deficiency affected chemokine receptor expression and B cell navigation, we isolated B cells from the BM of NLRP3−/− C56BL/6 mice and control mice and investigated the expression of CXCR4 and CCR7, two chemokine receptors important for B cell homing and positioning (Cyster, 1999). The proportions of B cells expressing both receptors were increased in NLRP3−/− mice in comparison with WT B cells (Fig S5). Interestingly, B cells isolated from mutant mice expressed significantly higher CXCR4 and CCR7 levels compared with control mice (Fig 5), suggesting that chemokine receptor overexpression contributed to their navigation to secondary lymphoid organs of mice lacking NLRP3. Thus, it is likely that the genetic ablation of *nlrp3* led to the up-regulation of chemokine receptor gene expression in B cells and that NLRP3 acts on B cells through chemokines and chemokine receptors in regulating immune cell homing and retention to lymphoid organs.

## Discussion

In the mammalian BM, B lymphocytes develop from pluripotent hematopoietic stem cells through an ordered process that involves differential expression of cell surface markers, sequential rearrangement of Ig H- and L-chain loci, and expression of stage-specific genes (Melchers, 2015). To amplify Ab diversity and to render B cells tolerant to self-antigens, receptor editing acts by displacing a productively rearranged Ig H- or L-chain gene by secondary gene rearrangements, forming edited BCRs with no or reduced autoreactivity (Radic & Zouali, 1996). Signals that coordinate these mechanisms are governed by dynamic biological processes that involve the BCR, ligands, cytokine receptors, and a complex network of transcription factors, including IRF4 and IRF8. In addition to limiting pre-B cell expansion, these transcription factors modulate L-chain rearrangement and transcription. IRF4 is known to bind motifs in L-chain gene enhancers and to enhance the transcription of target genes (Eisenbeis et al, 1995). As a result, encounter of immature B cells with self-antigen triggers rapid expression of IRF4 followed by secondary variable gene rearrangements and receptor editing, and this process is defective in IRF4-deficient mice (Pathak et al, 2008; Cadera et al, 2009). Herein, we found that ablation of *nlrp3* led to decreased numbers of *κ*-positive B cells and overexpression of IRF4 in B cells. Based on the fact that NLRP3 can interact with IRF4 in the nucleus of lymphocytes and that this binding can transactivate gene promoters (Bruchard et al, 2015), we envision that NLRP3 plays a functional role in the transcriptional network that regulates B cell development. In the absence of NLRP3, IRF4 expression is up-regulated, which results in enhanced initiation of k-chain rearrangements at the expense of Igλ-chain assembly.

Throughout B cell compartment, the V(D)J recombination machinery establishes allelic and isotypic exclusion at the Ig loci, a tightly regulated process that enables expression of a unique H- and L-chain pair and, consequently, a unique BCR in each lymphocyte (Vettermann & Schlissel, 2010). However, a small proportion of B cells expressing two types of L-chains, or less frequently H-chains, is detectable in both humans and mice. These allelicaly and isotypicaly included B cells represent less than 5% of the peripheral B cell pool (Casellas et al, 2007). In healthy mice, dual κ- and κ/λ-positive B cells have been shown to accumulate in the MZ B cell population of the spleen (Li et al, 2002). In the current studies, we observed that mice lacking NLRP3 exhibit a reduction of B cells expressing dual κ- and λ-light chains. It is possible that the overexpression of IRF4, known to bind motifs in L-chain gene enhancers and to promote the transcription of target genes (Eisenbeis et al, 1995), led to increased L-chain rearrangements that limited the production of isotypicaly included B cells.

Mature spleen B cell subsets exhibit different anatomical locations and phenotypes and play distinct functions. Previous work revealed that the MZ represents an important site of entry and exit of B cells from the spleen. Because of their low potential to adhere to stromal cells, FO B cells navigate from the follicle to the MZ before their egress to the bloodstream through the red pulp (Arnon et al, 2013). Although the pathways underlying their speciation have not been fully deciphered, several observations indicate that signals generated through the B cell surface are differentially interpreted in the nucleus by transcription factors that influence MZ versus FO lineage commitment. In particular, B cell fate dynamics is controlled by a mutual antagonism between the transcription factors IRF4 and IRF8 (Xu et al, 2015). For example, mice lacking the *irf4* gene manifest B cell accumulation in the MZ area, suggesting that IRF4 regulates the expression of genes important for MZ B cell trafficking and B cell positioning in the spleen environment (Simonetti et al, 2013). Here, we found that deficiency in NLRP3 resulted in up-regulation of IRF4 and reduction in MZ B cell numbers. We propose that NLRP3 plays a role in the controlling the IRF4-mediated transcriptional network that controls B cell fate decisions in the spleen. In the absence of NLRP3, the migration and homing properties of B cells are disrupted, thereby leading to aberrant positioning of B cell subsets in the spleen microenvironment.

In mice, B-1 cells normally reside in the peritoneum and the pleural cavity, but they also can navigate through secondary lymphoid organs, including the spleen or draining lymph nodes (Ghosn et al, 2019). They exert innate-like B functions by providing immune responses to pathogens and producing natural Abs that contribute to maintaining immune homeostasis (Baumgarth et al, 2000; Viau & Zouali, 2005). Typically, B-1 cells respond preferentially to Toll-like receptor signaling rather than to BCR ligation. Whereas the major B-1a subset expresses the CD5 marker, the minor B-1b subset is CD5-negative and is able to recognize a broader range of epitopes. Here, we demonstrated that NLRP3-deficient mice show an expansion of B-1 cells in the peritoneal cavity. Importantly, this loss does not affect B-2 cells that also reside at this anatomical location. We propose that the NLRP3 pathway is uniquely involved in generating and/or maintaining the B-1 pool, particularly the B-1a subset. First, it is possible that a functional NLRP3 pathway promotes generation of survival factors able to counteract apoptotic B cell death in the peritoneal cavity. Consistent with this view, IL-1, which can be generated as a result of NLRP3 activation, can synergize with other ligands to trigger B cell proliferation (Falkoff et al, 1983). Second, survival and self-renewal of B-1a cells in the highly lipid-rich peritoneal microenvironment requires a unique metabolic program that includes lipid uptake, predominant fatty acid synthesis, and high levels of glycolysis, and this metabolic pattern is likely to contribute to the potential of B-1 cells to respond rapidly to various stimulations compared with B-2 B cells (Clarke et al, 2018). Previous studies demonstrated that NLRP3 can govern adipocyte differentiation and function and acts as an important regulator of adipose tissue metabolism (Finucane et al, 2019), providing a potential mechanistic link between NLRP3 activation, metabolism, and B cell development in the peritoneal cavity. For example, differentiation of pre-adipocytes isolated from NLRP3−/− mice results in more metabolically active fat cells, and the mutant mice are resistant to the development of high-fat, diet-induced obesity, and their energy expenditure is enhanced (Stienstra et al, 2011). In our studies, it is plausible that genetic ablation of *nlrp3* promoted insulin signaling in adipocytes, leading to diminished glucose uptake and lipogenesis, which resulted in B-1 cell expansion. Third, positioning of lymphocyte subsets throughout lymphoid organs also relies on discrete sets of chemokines and their cognate receptors expressed in distinct niches. Chemokine receptors that regulate trafficking and retention of B cells include CXCR4 and CCR7 (Cyster, 1999). Their respective ligands (CCL19, CCL21, and CXCL12) produced by endothelial cells in lymphoid and non-lymphoid tissue and in body cavities regulate lymphocyte homing to and within secondary lymphoid organs. Mice lacking CCR7 exhibit a reduction in B-1 cells as a result of impaired egress of CCR7-deficient lymphocytes (Hopken et al, 2004). In the current studies, we found that genetic ablation of *nlrp3* led to up-regulation of chemokine receptor gene expression in B cells, which most likely promoted their homing to and retention in the peritoneal cavity.

Contemporary understanding of the functions of NLRP3 derives essentially from studies of innate immune cells, and its role in the adaptive branch of immunity has not been fully elucidated. Early studies indicated that mice lacking NLRP3 exhibit deficient Ab production (Kumar et al, 2009), which suggested that NLRP3 activation may regulate IgM secretion. Our present findings indicate that imbalanced expression of inflammasomes could have a significant impact on the life span of various B cell subsets and, consequently, the effectiveness of B cell responses. Our observations made in mutant mice could have implications for human pathology in patients with defects in inflammasome components, particularly because the specialized MZ B cell subset produces polyreactive Abs that impart immune protection to newborns and infants. The detailed mechanisms underlying the role of NLRP3 in retention and/or survival of MZ B cells remain to be fully deciphered.

By uncovering new functions of NLRP3 in B cells, our present findings, together with other T cell studies (Bruchard et al, 2015; Arbore et al, 2016; Javanmard Khameneh et al, 2019; Park et al, 2019), underscore the importance of a tightly regulated control of NLRP3 expression in T and B lymphocytes. Investigating the role of inflammasomes in the development of humoral immunity provides a further dimension to our understanding of the multifactorial nature of the immune system that could open new research avenues for potential therapy, particularly because NLRP3 plays a key role in various types of sterile autoinflammation.

## Materials and Methods

### Animals

Female C57BL/6 (B6) mice aged 5–6 wk were acclimated for 1–2 wk, and NLRP3-deficient mice (B6.129S6-Nlrp3tm1Bhk/J; Jackson Laboratory) were maintained at the experimental animal facility of China Medical University in accordance with the regulations of the Taiwan accreditation of laboratory animal care (Kovarova et al, 2012). After euthanasia, BM cells and splenocytes were isolated from each mouse as described previously (Viau et al, 2005). After red blood cell lysis by the EasySep buffer (STEMCELL Technologies) and washing, cells were suspended in culture medium for counting.

### B cell isolation

B cells were isolated from the BM and spleen by negative selection using the MagniSort Mouse B cell Enrichment Kit (Cat. 14-0161-82; Thermo Fisher Scientific) according to the manufacturer’s protocol. The purity of the isolated cells was routinely above 95%, as assayed by flow cytometry. B cells were isolated from the peritoneal cavity as described elsewhere (Ray & Dittel, 2010). The resulting untouched, negatively selected B cells were grown in RPMI culture medium (Gibco), containing 10 mM HEPES (HyClone), 1 mM sodium pyruvate (HyClone), MEM non-essential amino acids (HyClone), 10% heat-inactivated FBS (HyClone), 1% penicillin-streptomycin-amphotericin B solution (Biological Industries), and were maintained at 37°C in a 5% CO_2_ atmosphere.

### Flow cytometry staining and analysis

To prevent binding of conjugated antibodies to FcγR, purified B cells (2 × 10^5^) were first incubated with a monoclonal Ab that specifically recognizes a common non-polymorphic epitope on the extracellular domains of the mouse FcγIII (CD16) and FcγII (CD32) (Cat. 14-0161-82; Thermo Fisher Scientific) in 2% FBS for 10 min. After washing, B cells were stained with fluorescent-labeled antibodies for 30 min and fixed with 4% paraformaldehyde. The following labeled antibodies purchased from Biolegend, Inc., are specific for the mouse κ-light chain, λ-light chain, CD45R/B220, IgM, CD23, CD21, CD45R/B220, CD86, CD69, CXCR4, CCR7, CD11b, and CD5. Data were acquired on a BD FACS Celesta Cell Analyzer and analysis was conducted with FlowJo software.

### B cell proliferation assay with a tracking dye

Purified B cells were incubated with 1 mM CFSE tracking dye (Molecular Probes) in PBS at 37°C for 8 min in the dark. After adding a FBS stop solution and centrifugation, the stained cells were plated in a 96-well plate at 5 × 10^5^ cells/well and exposed to various stimuli. After 3 d of culture, B cells were analyzed by FACS. Data were acquired on BD FACS Celesta and analyzed with FlowJo software.

### Measurement of natural Ab production

Natural Ab production was measured by ELISA. Polystyrene microtiter plates were first coated with ENA solution (Cat. MBS319513; MyBioSource) diluted 100-fold in TBS overnight at 4°C, and blocked with 1% BSA in PBS for 2 h at room temperature. Serum samples diluted in PBS/BSA were added to the wells. After washing, bound Ab was detected with anti-IgM Ab labeled with HRP and diluted in 0.05 M carbonate buffer. Absorbance was recorded at 405 nm. Production of IgM Ab to single stranded DNA was assessed by ELISA using polystyrene microtiter plates irradiated with UV overnight, as described elsewhere (Zouali & Stollar, 1986).

### Quantification of gene expression levels by qRT–PCR

To test IRF4 and IRF8 expression, RNA was extracted from purified B cells using the RNeasy mini kit (Cat. 74106; QIAGEN), according to the manufacturer’s instructions, and expression of IRF4 and IRF8 in the cell lysates was analyzed by reverse transcription and qRT–PCR. First-strand cDNA templates were synthesized using total RNA from various samples using The iScript gDNA Clear cDNA Synthesis Kit (Cat. 1725035; Bio-Rad) following the manufacturer’s recommendations. The cDNA obtained served as the templates for a dye-based quantitative PCR (qRT-PCR) using the iQ SYBR Green Supermix kit (Cat. 1708882; Bio-Rad). The primers used for IRF4 amplification were forward: GTGGAAACACGCGGGCAAGC and reverse: GGCTCCTCT CGACCAATTCCTCA. For IRF8 amplification, we used forward: AGAGGGAGACAAAGCTGAACCAGCC and reverse: CCACGCCCAGCTTGCATTTT primers. For GAPDH, we used: forward: TGTGTCCGTCGTGGATCTGA and reverse: CCTGCTTCACCACCTTCTTGAT primers. Relative levels of expression of IRF4 and IRF8 mRNA were determined by the 2−ΔΔCt method.

### Statistical analysis

*t* test was used to determine the statistical significance of the data. A *P*-value < 0.05 was considered significant.

## Data Availability

The datasets used and/or analyzed during the current study are available from the corresponding author, who has the ORCID identifier 0000-0002-8225-456X, on reasonable request.

## Supplementary Material

Reviewer comments
